# The value of EYA1/3/4 in clear cell renal cell carcinoma: a study from multiple databases

**DOI:** 10.1038/s41598-023-34324-3

**Published:** 2023-05-08

**Authors:** Taobin Liu, Jianqiang Nie, Xiaoming Zhang, Xinxi Deng, Bin Fu

**Affiliations:** 1 Department of Urology, the First Affiliated Hospital of Nanchang University, Yong Wai Zheng Street 17#, Nanchang, 330006 Jiangxi Province People’s Republic of China; 2 Nanchang County People’s Hospital, 199 Xiangyang Road, Liantang Town, Nanchang County, Nanchang City, 330200 Jiangxi Province People’s Republic of China; 3 Department of Urology, Jiu Jiang NO.1 People’s Hospital, Jiujiang, 332000 Jiangxi Province People’s Republic of China

**Keywords:** Cancer, Genetics

## Abstract

There is evidence from multiple studies that dysregulation of the Eyes Absent (EYA) protein plays multiple roles in many cancers. Despite this, little is known about the prognostic significance of the EYAs family in clear cell renal cell carcinoma (ccRCC). We systematically analyzed the value of EYAs in Clear Cell Renal Cell Carcinoma. Our analysis included examining transcriptional levels, mutations, methylated modifications, co-expression, protein–protein interactions (PPIs), immune infiltration, single-cell sequencing, drug sensitivity, and prognostic values. We based our analysis on data from several databases, including the Cancer Genome Atlas database (TCGA), the Gene Expression Omnibus database (GEO), UALCAN, TIMER, Gene Expression Profiling Interactive Analysis (GEPIA), STRING, cBioPortal and GSCALite. In patients with ccRCC, the EYA1 gene was significantly highly expressed, while the expression of EYA2/3/4 genes showed the opposite trend. The level of expression of the EYA1/3/4 gene was significantly correlated with the prognosis and clinicopathological parameters of ccRCC patients. Univariate and multifactorial Cox regression analyses revealed EYA1/3 as an independent prognostic factor for ccRCC, establishing nomogram line plots with good predictive power. Meanwhile, the number of mutations in EYAs was also significantly correlated with poor overall survival (OS) and progression-free survival (PFS) of patients with ccRCC. Mechanistically, EYAs genes play an essential role in a wide range of biological processes such as DNA metabolism and double-strand break repair in ccRCC. The majority of EYAs members were related to the infiltration of immune cells, drug sensitivity, and methylation levels. Furthermore, our experiment confirmed that EYA1 gene expression was upregulated, and EYA2/3/4 showed low expression in ccRCC. The increased expression of EYA1 might play an important role in ccRCC oncogenesis, and the decreased expression of EYA3/4 could function as a tumor suppressor, suggesting EYA1/3/4 might serve as valuable prognostic markers and potential new therapeutic targets for ccRCC.

## Introduction

Kidney cancer incidence and mortality rates are rising and a total of 73,750 new cases are expected by 2021 in the United States^1^. Renal carcinoma, which accounts for 90–95% of kidney cancer cases, is the most frequently diagnosed type of kidney cancer in adults^2^. Renal cell carcinoma (RCC), commonly known as renal carcinoma, is a prevalent malignancy of the urinary tract and is responsible for 2–3% of all cancer cases^3^. Renal cell carcinoma (RCC), the most common type of kidney cancer, can be classified into three major subtypes based on their cell appearance: clear cell renal cell carcinoma (ccRCC), papillary renal cell carcinoma (pRCC), and chromophobe renal cell carcinoma (chRCC). Among them, ccRCC accounts for 70–80% of all RCC cases, while pRCC and chRCC account for 10–20% and 5% of RCC cases, respectively^4^. RCC is characterized by high angiogenic and hypoxic conditions, as well as limited sensitivity to chemotherapy, rendering surgical resection the most effective approach to managing localized RCC^5^. Patients with renal carcinoma have a poor prognosis because most have already examined distal metastases during the initial diagnosis or after the primary tumor has been removed^6,7^. In general, around one-third of kidney cancer patients are diagnosed with metastatic disease, while 40% of patients with locally advanced disease are at a high risk of disease recurrence following surgical resection^8,9^. In recent years, targeted agents and immunotherapies have made a great deal of progress in treating metastatic RCC. However, the emergence of drug resistance is a major challenge for current cancer treatments, forcing us to reconsider how to treat RCC^10^. This underscores the significance of timely diagnosis and intervention for individuals affected by RCC. We must find a novel prognostic biomarker and a potential therapeutic target by further elucidating the underlying mechanisms of the development of kidney cancer.

Initial studies identified the eye absent (EYA) proteins as essential coactivators of six families of homeoprotein's^11^, which were required for normal eye development in Drosophila^12,13^. In mammals, the EYA family comprises four members (EYA1/2/3/4), each of which contains a C-terminal EYA domain (ED) that is highly conserved, as well as an N-terminal transactivation domain that is relatively less conserved. The EYA gene family encodes proteins with a variety of functions that function as transcriptional coactivators, as well as Tyr phosphatases of the haloacid dehalogenase family and EYA proteins, which are involved in EGFR/Ras/MAPK, the Notch signaling pathway, Wingless, and Hedgehog pathways and so on. Current studies suggest that activation of EYA phosphatases contributes to the invasion, migration, and transformation of tumor cells and that these processes are mediated by altering the actin cytoskeleton^14^. The abnormal function of EYA family genes has been revealed to play critical roles in tumorigenesis and progression^15^. Study findings suggested that The EYA1 protein promotes the migration and invasion of hepatocellular carcinoma (HCC) by activating the FNDC3B protein^16^. Six1 promoted breast cancer metastasis in mouse models through Six1/Eya interaction, which ED mediated^17,18^. A group of researchers has reported that elevated levels of EYA2 mRNA were observed in ovarian cancer and that the expression of EYA2 was correlated with tumor progression^19^. Researchers have shown that EYA3, an oncogene, sensitizes Ewing sarcoma cells to DNA-damaging chemotherapy^20^. In vivo, studies have demonstrated the impact of EYA on cellular invasiveness, with the silencing of EYA3 expression resulting in the inhibition of metastasis in the MDA-MB-231 invasive breast cancer cell line^14^. Recent studies have shown that abnormal methylation of the EYA4 gene may be a biomarker for colorectal cancer, urothelial bladder cancer, and breast cancer^21,22^. These data indicated that the EYA family members function as tumor oncogenes or suppressor genes in the occurrence and progression of tumors in human cancers. Thus far, limited knowledge exists regarding the involvement of the EYA family in ccRCC, and further clarification is needed to fully understand the expression patterns and prognostic significance of EYAs in ccRCC.

In this study, we performed an analysis of thousands of published gene expression and copy number variation datasets to investigate the expression patterns, functional roles, and prognostic significance of the EYA family members in patients with ccRCC.

## Materials and methods

### Tumor samples and patient

Tumor samples were obtained from 10 patients, who were diagnosed with kidney renal clear cell carcinoma. Ten pairs of ccRCC and their corresponding adjacent normal tissues were subjected to pathological confirmation and independent validation by two pathologists. All ccRCC tissues and adjacent normal kidney tissues used in this study were obtained from the Department of Urology of the First Affiliated Hospital of Nanchang University between 2021 and 2022 and stored in liquid nitrogen.

### RNA extraction and qRT-PCR

We extracted total RNA, produced complementary DNA (cDNA), and performed a polymerase chain reaction. Details of the procedure and primer sequences were as follows:

- Human EYA1 forward primer: TGTTGGAGGTCTGCTTGGTC, Human EYA1 reverse primer: TGAGCGAGAGTGCTTTCAGG;
- Human EYA2 forward primer: GTGGTGATCGGTGATGGTGT, Human EYA2 reverse primer: GAGATGCTGCTGATCCTGCT;
- Human EYA3 forward primer: CAGCAGTAGCCAGCATCTCA, Human EYA3 reverse primer: GGTGCTCTCTGCATCACTGT;
- Human EYA4 forward primer: AGCGTGTGTTTGTCTGGGAT, Human EYA4 reverse primer: TCTTCCATGCGGAGTCCAAG;
- Human GAPDH forward primer GCCACATCGCTCAGACACCAT, Human GAPDH reverse primer: CCCATACGACTGCAAAGACCC.

SYBR Real-Time PCR kit (USA) from Qiagen was used for the qRT-PCR under the following conditions:95 °C for 1 min, followed by 40 cycles of 95 °C for 5 s and 65 °C for 10 s. The internal control is Glyceraldehyde-3-phosphate dehydrogenase (GAPDH).

### Predictive modeling and validation

Sample of 541 ccRCCs from the public database TCGA was collected and analyzed using R software (proportional risk hypothesis testing and Cox regression analysis using the “survival” package, and nomogram correlation model construction and visualization using the “rms” package). Calibration curves were used to determine the accuracy of the nomogram model predictions. External validation was performed using the ArrayExpress database (E-MTAB-1980) (https://www.ebi.ac.uk/services).

### UALCAN

The UALCAN web portal provides users with the ability to explore the relationship between candidate genes and clinical features of tumors. The portal offers RNA-seq and clinical data for 31 types of cancer based on TCGA^23^. Analyses of the expression levels of EYAs were conducted in this study in normal tissues and ccRCC tissues. Additionally, the promoter methylation levels of EYAs in normal tissues and those of patients with ccRCC were studied.

### GEPIA

The GEPIA dataset (www.gepia.cancer-pku.cn) contains a spectrum of cancer expression data^24^. The GEPIA dataset includes 9,736 tumor samples and 8,587 normal samples collected from the TCGA. As part of this study, we analyzed EYAs gene expression levels in ccRCC tissues and normal tissues using the GEPIA database.

### cBioPortal

The cBioPortal (www.cbioportal.org) provides multidimensional visualization and analyses of cancer genomics data from the TCGA database^25^. In our study, we analyzed 512 renal cancer samples (TCGA provisional). The samples contained gene mutations, copy number alterations (CNA) from GISTIC, mRNA expression Z-score (RNA Seq V2 RSEM), and protein expression Z-score (RPPA). Genetic alterations, co-expression, and network modules of EYAs were obtained from cBioPortal. In addition, genetic mutations in EYAs genes were correlated with OS and disease-free survival (DFS) of ccRCC patients. We conducted a log-rank test to evaluate whether there were differences between the altered and unaltered groups.

### STRING

The STRING database (https://string-db.org/) compiles, assesses, and combines publicly available PPI data and augments them with computational forecasts of potential functions. We constructed a PPI network to investigate the interactions between EYAs and the top 50 frequently altered neighboring genes.

### TIMER

TIMER (cistrome.shinyapps.io/timer) is a tool that enables researchers to comprehensively examine tumor-immune interactions. With TIMER, users can utilize six main analytical modules to explore the correlation between immune infiltrates and various factors such as gene expression, clinical outcomes, somatic mutations, and somatic copy number alteration (SCNA)^26^. In our study, we employed the gene module to display the relationship between EYAs mRNA levels and immune cell infiltration levels in ccRCC. The "SCNA module" allowed us to compare tumor infiltration levels among tumors with different SCNA for EYAs.

### TISCH2

Tumor Immune Single-cell Hub 2 (TISCH2) is an online platform for analyzing the tumor microenvironment with single-cell resolution^27^. TISCH2 (http://tisch.comp-genomics.org/home/) collects a large amount of RNA sequencing data at human/mouse single-cell resolution, including samples from different tissues, organs, and disease states. To date (March 1, 2023), the TISCH2 database has 190 datasets and information related to 6,297,320 cells. The database provides an intuitive interface that allows users to easily browse, search, and query RNA-seq data and select different samples and conditions for analysis according to their interests and needs. TISCH2 also offers a range of analysis tools and charts, including clustering analysis, gene expression heatmaps, differential gene analysis, enrichment analysis, etc., to help users explore the transcriptome changes and functions of human mononuclear cells in depth. We used the TISCH2 database to analyze the expression distribution of EYA3/4 members in immune cells in ccRCC tissues. In our study, we selected two single-cell sequencing datasets (KIRC_GSE111360 and KIRC_GSE159115) from the public database GEO (https://www.ncbi.nlm.nih.gov/geo/)^28,29^.

### GSCALite

GSCALite (http://bioinfo.life.hust.edu.cn/web/GSCALite/) is a comprehensive online tool for gene set and drug sensitivity analysis. By leveraging the Genomics of Drug Sensitivity database (GDSC) and the Therapeutics Response Portal (CTRP), GSCALite collects drug sensitivity data and gene expression profiles of cancer cell lines. In our study, we utilized the drug-sensitivity module of GSCALite to investigate the association between drug sensitivity and EYAs gene expression profiling data of cancer cell lines. Additionally, we employed the methylation module to analyze the correlation between methylation and gene expression.

### Statistical analysis

Statistical analyses were performed using R software (version 3.6.2). To analyze the different expressions of EYAs members in ccRCC, we used the “limma” R package and the Wilcox test. We performed Kaplan–Meier survival analysis and Cox proportional hazards regression analysis to determine the prognostic significance of EYAs. We applied univariate Cox regression analysis to assess the impact of clinicopathological factors and mRNA expression levels of EYAs on the survival of ccRCC patients. Variables with a *P*-value < 0.1 were subjected to subsequent analysis. All statistical tests were three-sided, and the statistical significance level was set at 0.05.

### Ethics approval and consent to participate

All patients provided written informed consent, and the study was conducted in compliance with the ethical principles outlined in the Declaration of Helsinki. The study was also approved by the Institutional Ethics Committee of First Affiliated Hospital of Nanchang University (Approval Number: 202012-110).

## Results

### Transcriptional levels different of EYAs in patients bearing ccRCC

**Figure 1:**
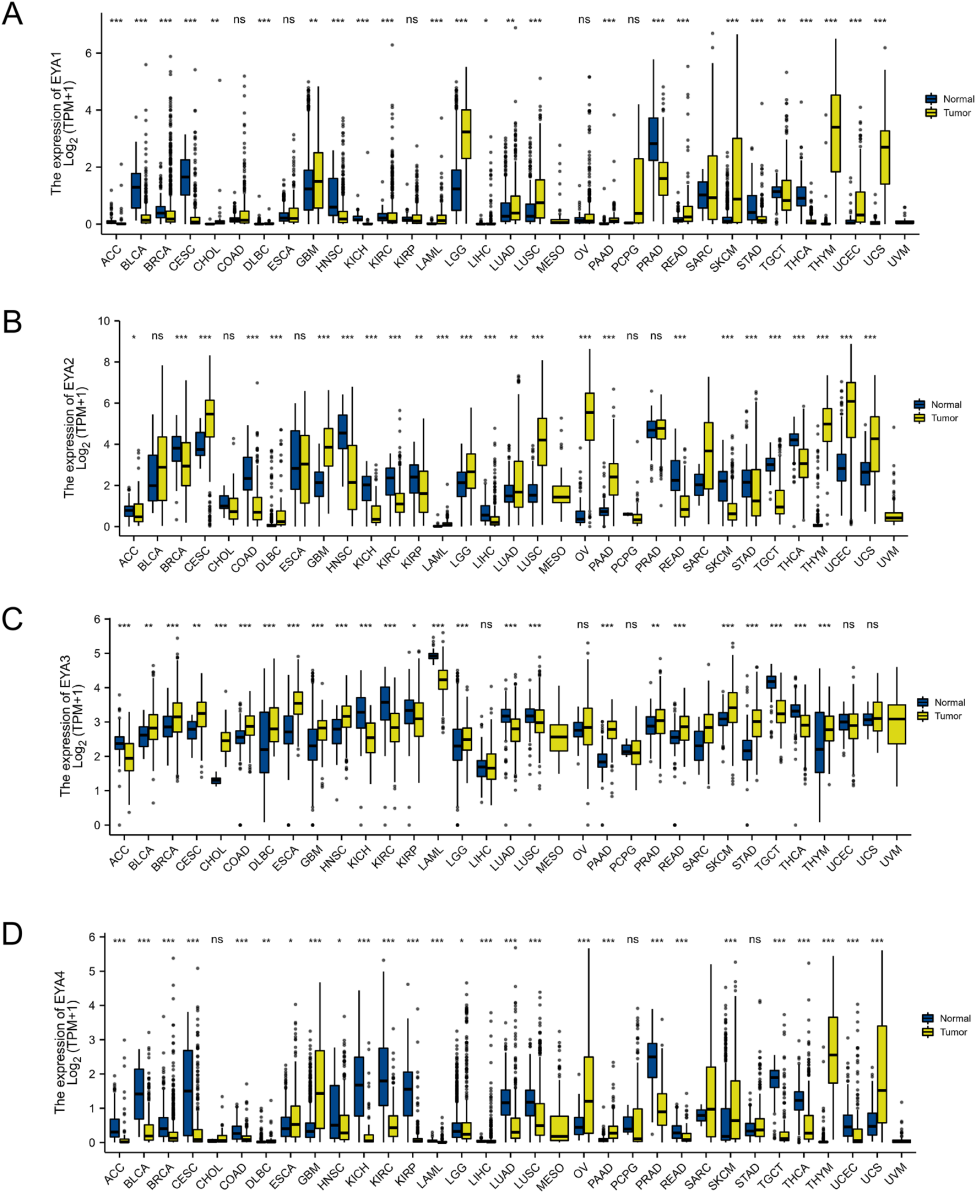
Pan-cancer EYA1 expression analysis (**A**). The mRNA expression of EYA2 in pan-cancer (**B**). The mRNA expression of EYA3 in pan-cancer (**C**). The mRNA expression of EYA4 in pan-cancer (**D**). ns indicates not significant; *P < 0.05; **P < 0.01; ***P < 0.001.

To begin, we explored the dysregulated transcriptional levels of the EYAs (EYA1, EYA2, EYA3, EYA4) family in 34 types of human common cancer. Based on the TCGA (http://portal.gdc.cancer.gov/) dataset, Fig. 1 was created with the packages ggplot2 (version 3.3.2) and R (version 3.6.1). As showed in Fig. 1A, the expression of EYA1 was significantly upregulated in 14 types of cancers including cholangiocarcinoma (CHOL), glioblastoma (GBM), colon adenocarcinoma (COAD), lymphoid neoplasm diffuse large B cell lymphoma (DLBC), acute myeloid leukemia (LAML), brain lower grade glioma (LGG), lung adenocarcinoma (LUAD), pancreatic adenocarcinoma (PAAD), lung squamous cell carcinoma (LUSC), rectum adenocarcinoma (READ), skin cutaneous melanoma (SKCM), uterine corpus endometrial carcinoma (UCEC), thymoma (THYM), and uterine carcinosarcoma (UCS) and downregulated in 12 types of cancers including adrenocortical carcinoma (ACC), bladder urothelial carcinoma (BLCA), breast invasive carcinoma (BRCA), cervical squamous cell carcinoma (CESC), kidney renal clear cell carcinoma (KIRC), head and neck squamous cell carcinoma (HNSC), prostate adenocarcinoma (PRAD), kidney chromophobe (KICH), liver hepatocellular carcinoma (LIHC), stomach adenocarcinoma (STAD), testicular germ cell tumors (TGCT) and testicular germ cell tumors (THCA). The expression of EYA2 was significantly upregulated in 12 types of cancers (CESC, DLBC, GBM, LAML, PAAD, LUAD, LUSC, LGG, OV, THYM, UCEC, and UCS) and downregulated in 12 types of cancers (CESC, ACC, BLCA, HNSC, READ, SKCM, KICH, LIHC, STAD, KIRC, TGCT, and THCA) (Fig. 1B). The expression of EYA3 was significantly upregulated in 17 types of cancers (HNSC, BLCA, BRCA, CESC, ESCA, GBM, COAD, DLBC, OV, PAAD, CHOL, LGG, PRAD, STAD, SKCM, READ, and THYM) and downregulated in 4 types of cancers (KIRC, ACC, KICH, LUAD, LUSC, KIRP, LAML, LIHC, TGCT, and THCA) (Fig. 1C). The expression of EYA4 was significantly upregulated in 6 types of cancers (ESCA, PAAD, GBM, OV, SKCM, and UCS), and downregulated in 13 types of cancers (LUAD, ACC, BLCA, BRCA, KIRC, HNSC, KIRP, CESC, COAD, DLBC, LUSC, KICH, LAML, LGG, LIHC, READ, THCA, PRAD, THYM, and UCEC) (Fig. 1D).

**Figure 2:**
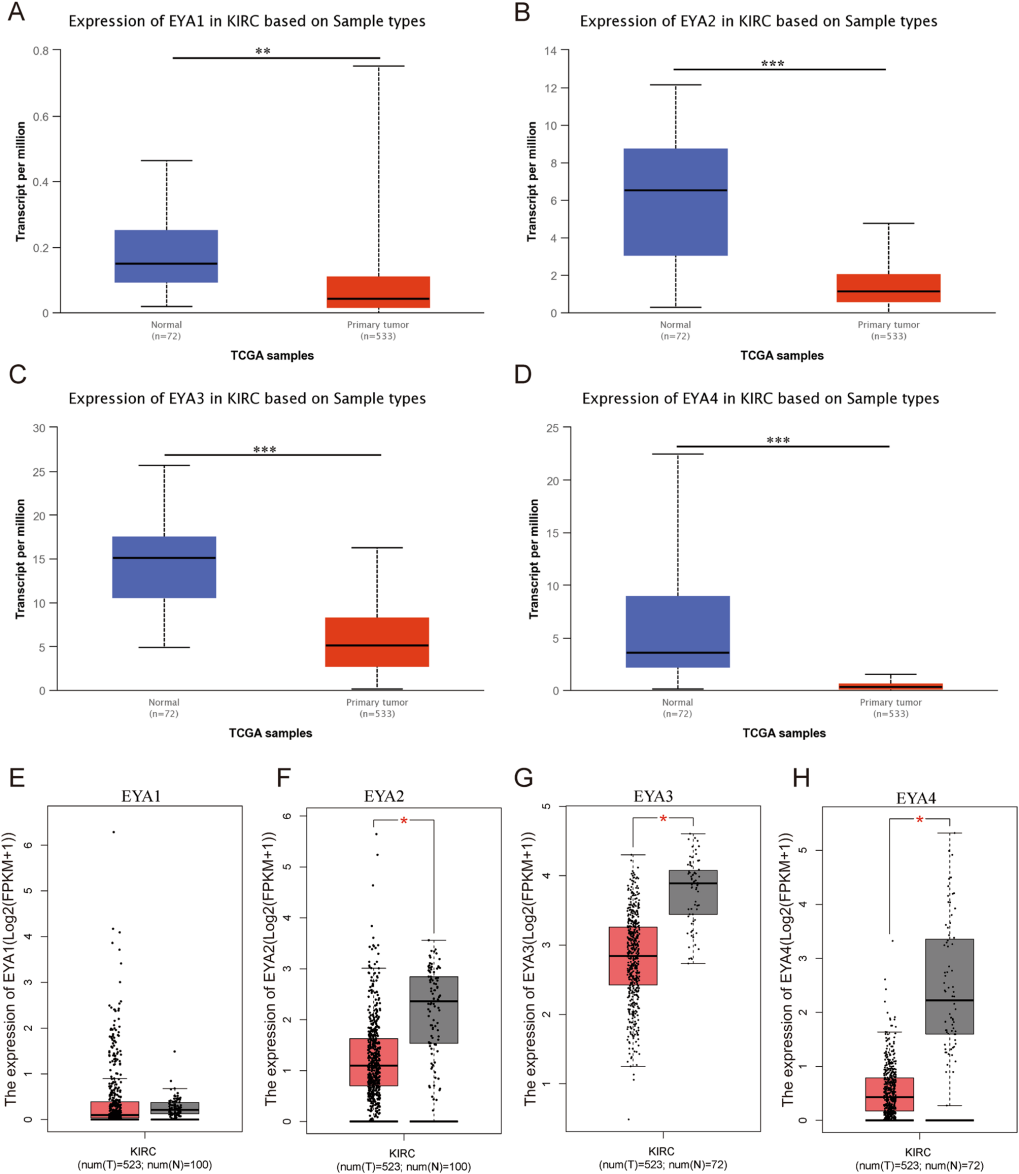
Different EYA1/2/3/4 family members are expressed in ccRCC and normal kidney tissues based on UALCAN (**A**, **B**, **C**, **D**) and GEPIA (**E**–**H**). *P < 0.05; **P < 0.01; ***P < 0.001.

**Figure 3:**
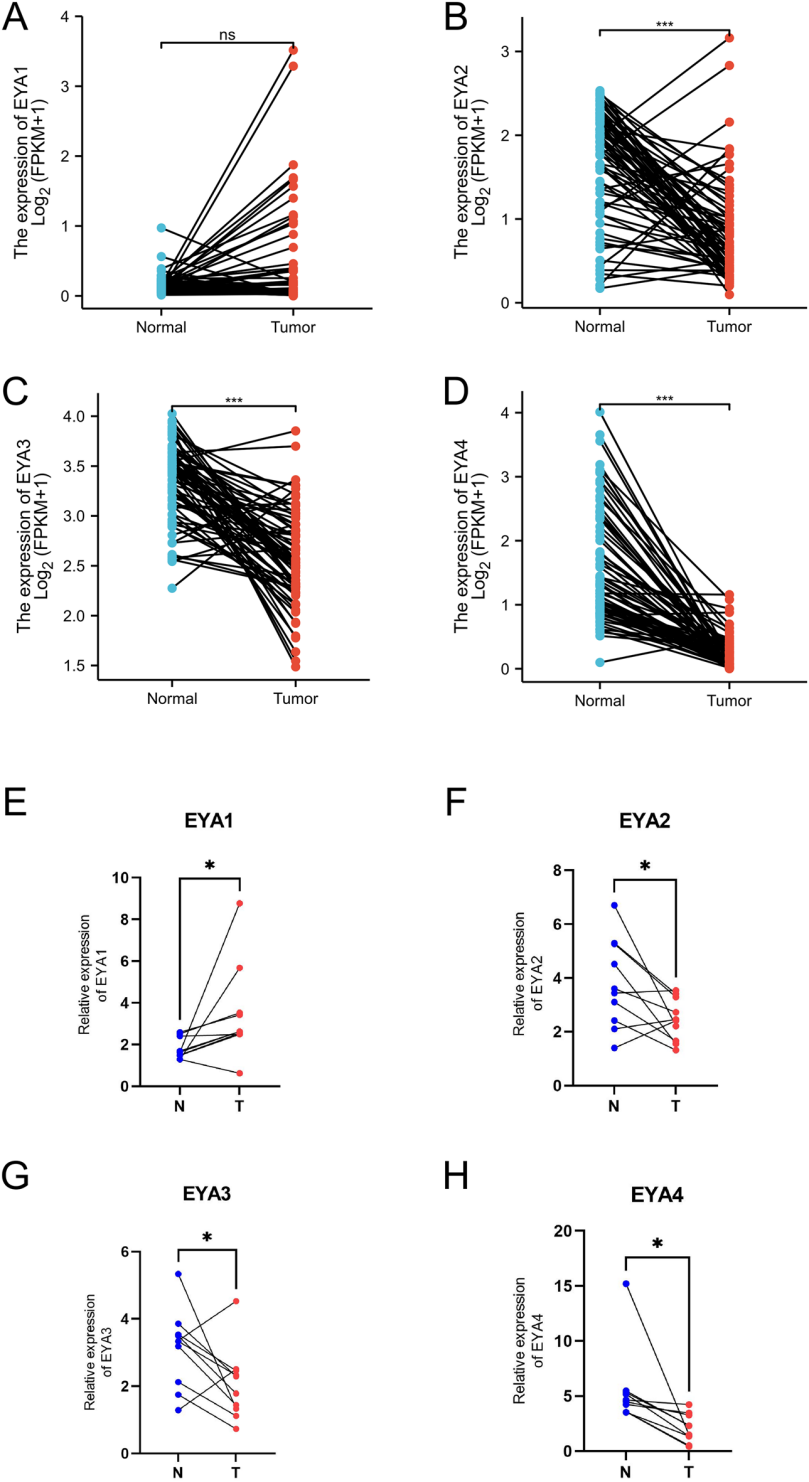
Transcriptional expression of EYA1/2/3/4 genes in 72 pairs of kidney cancer tissues and their matched normal adjacent tissues from the TCGA and GTEx databases (**A**–**D**). qRT-PCR analysis of EYA1/2/3/4 expression in ccRCC tissues and paired-adjacent normal kidney tissues (**E**–**H**). ns indicates not significant; *P < 0.05; ***P < 0.001.

Subsequently, we evaluated the transcription levels of the four EYA mRNAs in ccRCC patients according to the GEPIA database and the ULCAN cancer database (http://ualcan.path.uab.edu/index.html). As showcased in Fig. 2A–D, the transcriptional levels of EYA1, EYA2, EYA3, and EYA4 in ccRCC tissues were significantly decreased than in normal tissues. As can be seen in Fig. 2E–H, compared with non-cancerous normal tissues, low expressions of EYA2, EYA3, and EYA4 were observed in ccRCC tissues while there was no significant difference in EYA1 expression between cancer and noncancer tissues. Then, the expression levels of the EYAs family in 72 pairs of ccRCC samples and paired normal tissue samples were analyzed based on the TCGA dataset. As showcased in Fig. 3A–D, it was found that EYA 2/3/4 was transcriptionally expressed at significantly lower levels in ccRCC tissues (*P* < 0.05 for all) compared with normal tissues, while EYA1 did not differ significantly between renal cancer samples and normal samples. In addition, the relative mRNA expression levels of EYAs genes were verified by real-time qPCR among 10 paired ccRCC tissues and paired adjacent normal renal tissues. The level of mRNA of EYA1 was highly expressed in kidney cancer, while EYA2/3/4 was expressed low in cancer tissues with adjacent normal tissues (Fig. 3E–H, respectively). Considering the above results, we concluded that the transcriptional levels of EYA 2/3/4 in ccRCC tissues were expressed at significantly lower levels than those in paired normal tissue samples, while EYA1 exhibited the opposite result.

### Relationship between clinicopathological parameters and mRNA levels of EYAs in patients with ccRCC

**Figure 4:**
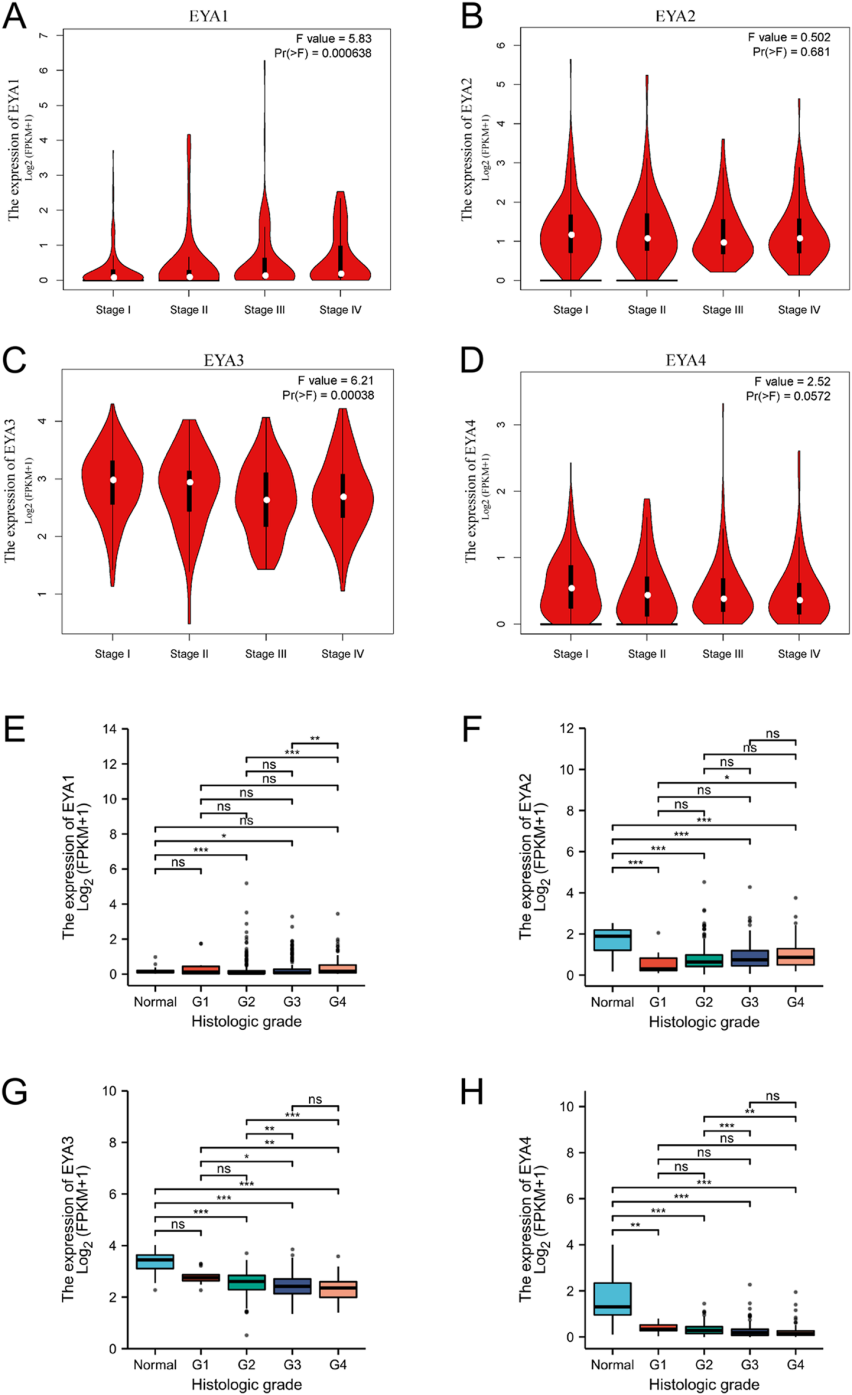
Correlations between EYAs mRNA levels and clinical stages in ccRCC patients were evaluated via violin plots using data from the GEPIA database. The mRNA expressions of EYA1 and EYA3 were significantly related to patients' pathological stages (**A**, **C**), whereas EYA2 and EYA4 were not (**B**, **D**). Associations of EYAs expressions with histologic grades of ccRCC were presented in **E**–**H**, respectively. ns indicates not significant; *P < 0.05; **P < 0.01; ***P < 0.001.

As a follow-up to our study of EYAs mRNA expression in normal samples and ccRCC tissues, we next analyzed of the TCGA data and GEPIA database to explore the correlation between the mRNA expression levels of EYAs and clinicopathological characteristics such as individual pathological stage and tumor grade. As showcased in Fig. 4A–D, the statistical analysis showed that the lower expression level of EYA1 and EYA3 was significantly correlated with the pathological stage, while the expression of EYA2/4 mRNA was not correlated with patients' pathological stage. It seemed that with increasing pathological stages of ccRCC, the expression of EYAs mRNA expressed lower, and the lowest mRNA expressions of EYAs were detected in stage III or stage IV. Further exploration of the relationship between EYAs and tumor grade revealed that there is a significant correlation between the expression of EYAs and tumor grade according to the TCGA data using R software version 3.6.3. The mRNA expression of EYAs showed a decreasing trend as the tumor grade increased. Notably, the lowest mRNA expression of EYA1/2 was observed in tumor grade 2 (Fig. 4E,F), whereas the lowest mRNA expression of EYA3/4 was found in grade 4 (Fig. 4G,H). Furthermore, as the tumor grade increased, a decrease in mRNA expression of EYA3/4 was observed. These findings indicate a significant correlation between the mRNA expression levels of all four members of the EYAs family and clinical and pathological parameters in ccRCC patients.

### Prognostic value of mRNA expression of EYAs in patients bearing ccRCC

**Figure 5:**
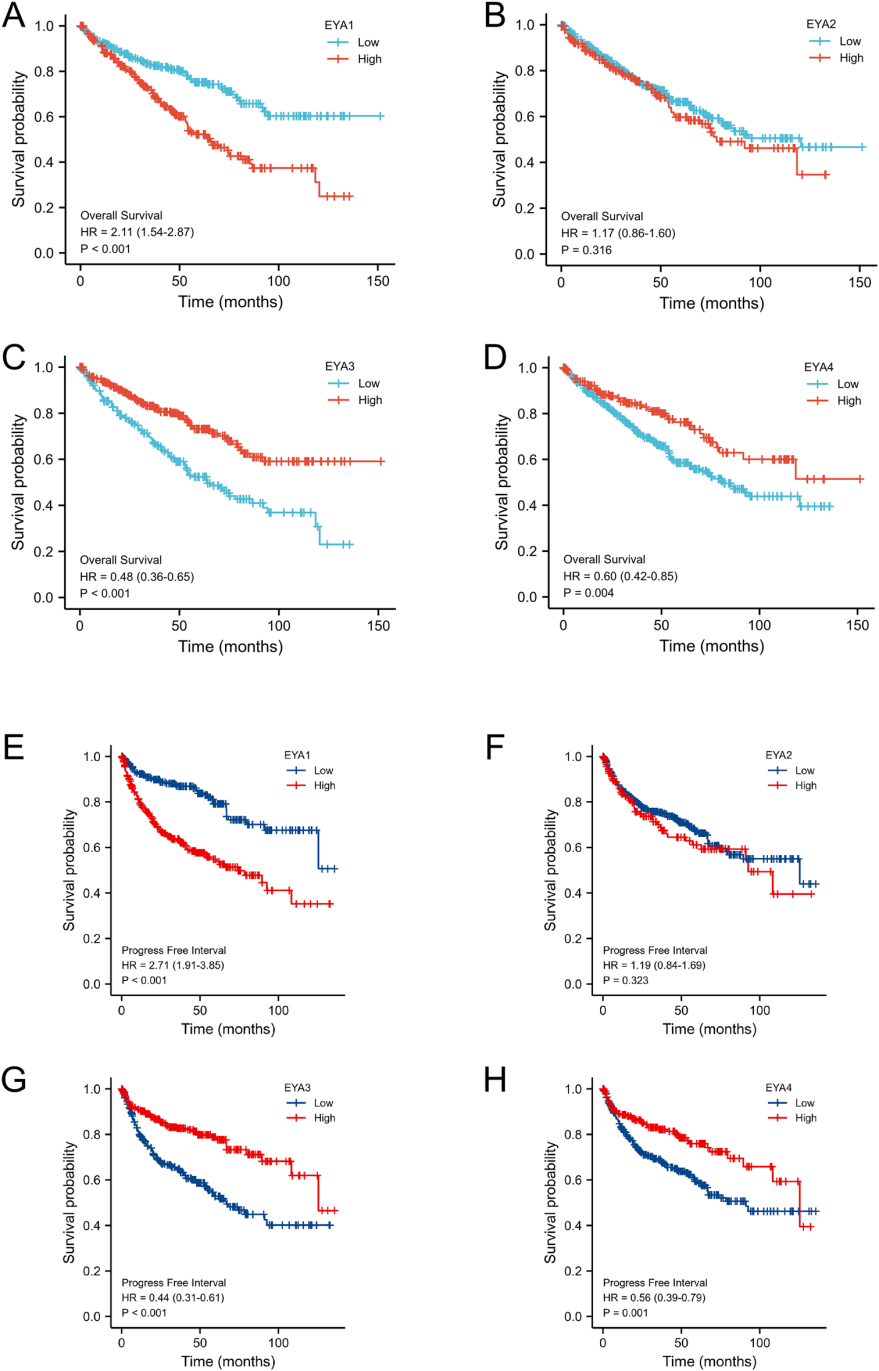
Prognostic value of EYAs mRNA expression levels in ccRCC. Relationships between EYAs mRNA expression levels and overall survival (OS) of ccRCC patients were conducted using R software based on TCGA data (**A**–**D**). Relationships between EYAs mRNA expression levels and progression-free survival (PFS) of ccRCC patients were analyzed using R software based on TCGA data (**E**–**H**).

Moreover, we analyzed the prognostic value of EYA mRNA expression with Kaplan–Meier survival curves in ccRCC patients based on TCGA ccRCC data. As was shown in Fig. 5A–D, our results showed that lower mRNA expression of EYA3 (HR = 0.48, 95%CI 0.36–0.65, and *P* < 0.001) and EYA4 (HR = 0.60, 95%CI 0.42–0.85, and *P* = 0.004) was closely associated with poorer overall survival (OS) in ccRCC patients, while a high level of EYA1 (HR = 2.11, 95%CI 1.54–2.87, and *P* < 0.001) mRNA expression was associated with poor OS. As was shown in Fig. 5E–H, a lower expression of mRNA for EYA3 (HR = 0.44, 95%CI 0.31–0.61, and *P* < 0.001) and EYA4 (HR = 0.56, 95%CI 0.39–0.79, and *P* = 0.001) was closely associated with a worse OS in ccRCC patients, while a high level of EYA1 (HR = 2.71, 95%CI 1.91–3.85, and *P* < 0.001) mRNA expression was associated with poor PFS. These results suggest that the mRNA expression of the EYA1/3/4 gene is significantly correlated with the prognosis of ccRCC patients and therefore can be used as a better biomarker to predict the survival time of ccRCC.

### EYAs family gene as an independent prognostic factor for OS in ccRCC patients

After identifying a significant correlation between mRNA expression of EYA1/3/4 and the prognosis of kidney cancer patients, we conducted a multivariate Cox regression analysis using the TCGA dataset to determine whether mRNA expression of EYA1/3/4 could independently predict patients' prognosis^30^. As a result of our univariate Cox analysis, we found that high mRNA expressions of EYA1 (HR = 2.362, 95%CI 1.726–3.231, and* P* < 0.001), and low mRNA expression of EYA3 (HR = 0.502, 95%CI 0.368–0.685, and* P* < 0.001) were related to shorter OS of ccRCC patients. A multivariate analysis of OS revealed that EYA1 (HR = 1.717, 95%CI 1.031–1.634, and* P* = 0.020) mRNA expression was independently related to shorter OS of patients with ccRCC and low EYA3 (HR = 0.517, 95%CI 0.328–0.814, and* P* = 0.004) mRNA expression was independently related to longer OS (Supplementary Table 1). In conclusion, the results imply that EYA1/3 transcriptional expression plays an independent prognostic role in clear cell renal cell carcinoma.

### Development and validation of the nomogram prognostic model

**Figure 6:**
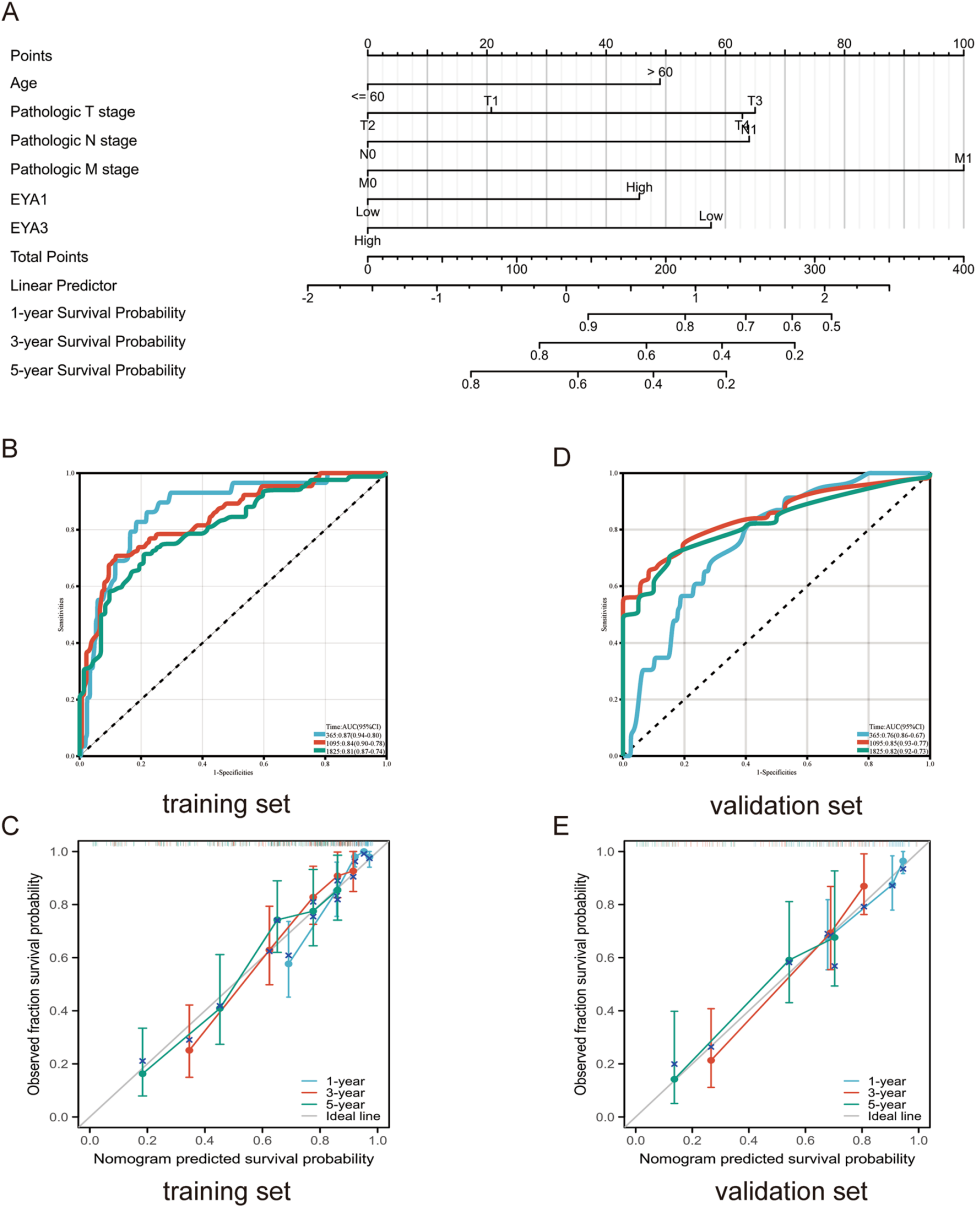
The nomogram prognostic model (**A**). ROC curve (**B**) and calibration curve (**C**) of the training set. ROC curve (**D**) and calibration curve (**E**) of the validation set.

The independent prognostic factors (including age, pathologic TNM stage, EYA1, and EYA3) after Cox regression analysis were selected, and a nomogram prognostic model was developed (Fig. 6A). A scaled line segment was used to integrate multiple predictors and plot them on the same plane at a certain scale to express the interrelationships among the predictor variables in the prognostic model. The AUC values of the ROC curves for 1, 3, and 5 years are 0.87 (0.94–0.80), 0.84 (0.0.90–0.78), and 0.81 (0.87–0.74), respectively (Fig. 6B). It is evident from the calibration curves of the prediction model that the 1-year, 3-year, and 5-year curves fit the diagonal better (Fig. 6C), indicating the high accuracy of the prediction model. Subsequently, the external validation using the E-MTAB-1980 database was also satisfactory (Fig. 6D,E).

### A study of the genetic mutation status in EYAs and their association with OS and PFS of patients with ccRCC

**Figure 7:**
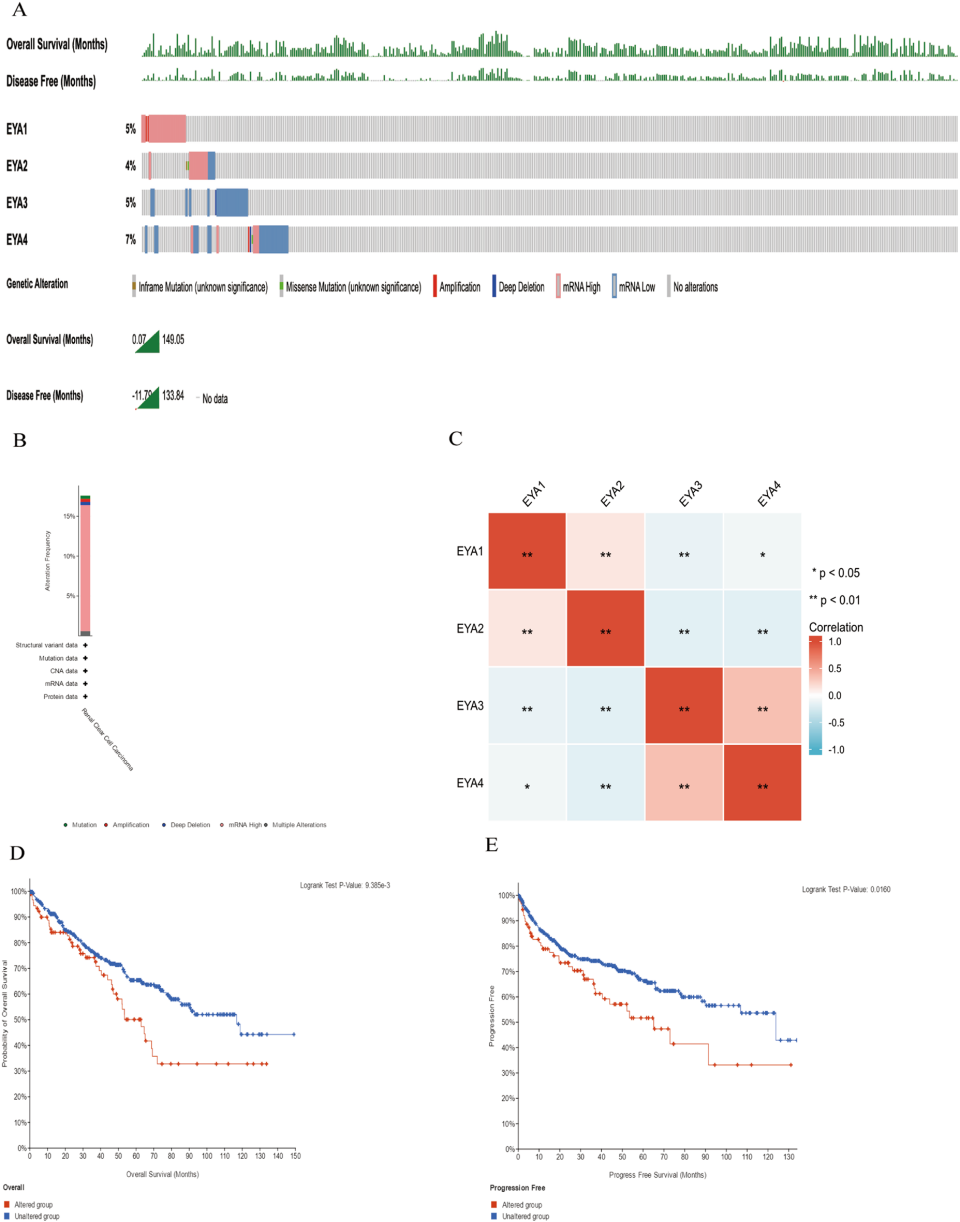
The genetic alterations of EYAs in ccRCC (**A**). Alteration frequency of EYAs according to the cBioPortal database (**B**). Correlation between four EYAs family members in ccRCC (**C**). Kaplan–Meier plots and log-rank tests revealed the overall survival of ccRCC patients with or without EYAs alterations (**D**) and progress free survival of ccRCC patients with or without EYAs alterations (**E**).

Through the use of the cBioPortal online tool, we assessed the association between genetic alterations in EYAs and OS, and PFS among patients with ccRCC. Fig. 7A presents the genetic alteration of EYAs in ccRCC, and Fig. 7B shows the frequency of genetic alteration according to the cBioPortal database. Among the 512 patients with ccRCC that were sequenced, a total of 90 patients were shown to have genetic changes, with a mutation rate of 18%. When compared to the other EYAs proteins, EYA1 exhibited the highest mutation rate, with four (8%) mutations detected. The mutation rate of EYA3/4 was 4% and the mutation rate of EYA2 was 2.9%. Additionally, we calculated the correlation between EYAs by analyzing their mRNA expression via the cBioPortal online tool. The results showed that EYA1 and EYA2 had a negative correlation with EYA3 and EYA4, while EYA4 was found to have a relatively strong correlation with EYA3 (Fig. 7C). Furthermore, the Kaplan-Meier plots and log-rank tests showed that genetic alteration in EYAs was associated with shorter OS (Fig. 7D, *P* = 9.385e-3) and DFS (Fig. 7E, *P* = 0.0160) in patients with ccRCC. Based on these results, genetic changes in EYAs could significantly influence the long-term prognosis of ccRCC patients as well.

### Predicted functions and pathways of mutations in EYAs and their 50 frequently altered neighbor genes in ccRCC patients

**Figure 8:**
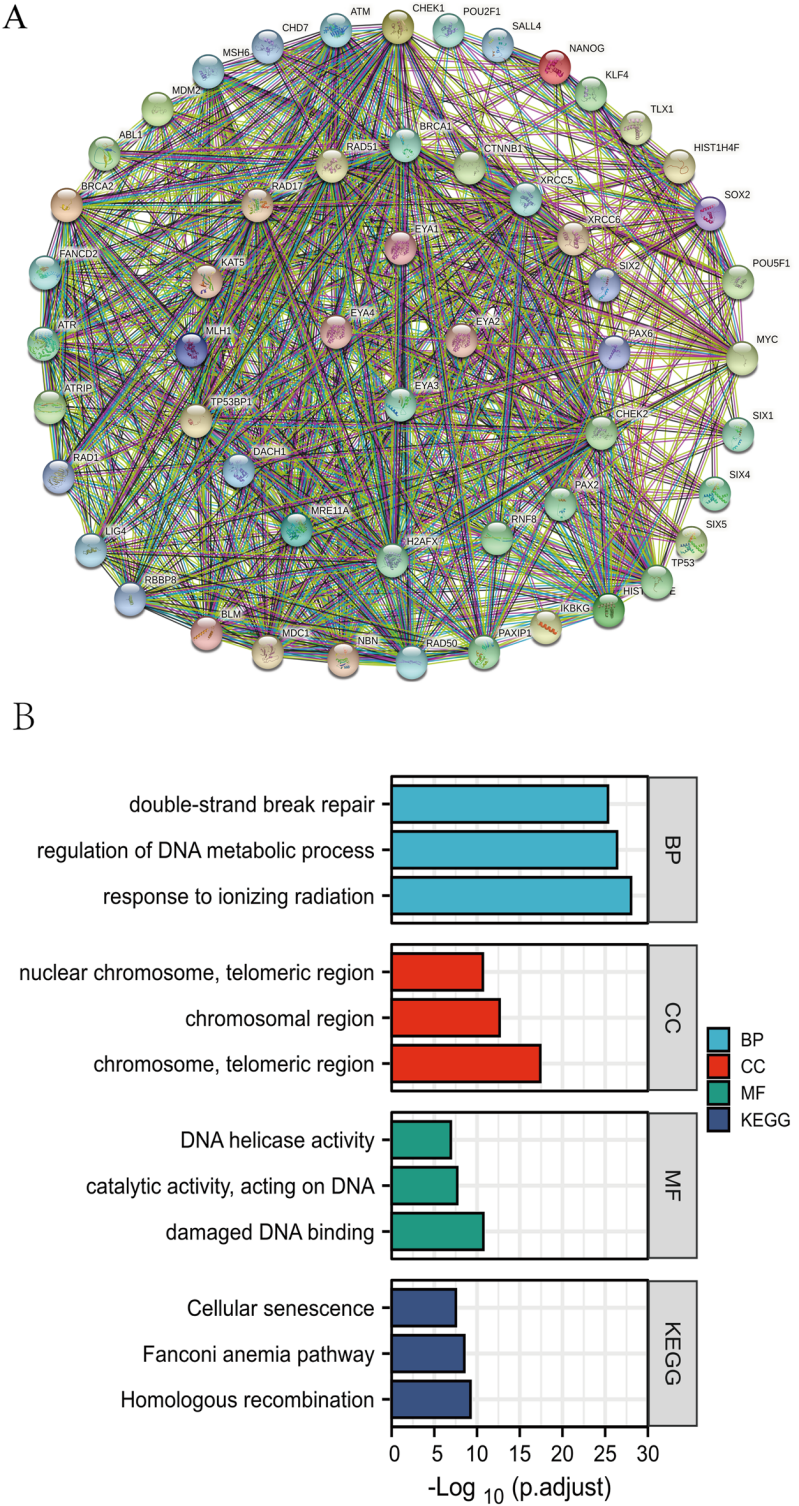
A protein–protein interaction (PPI) network of EYAs and the 50 neighboring genes associated with the mutations of EYAs in ccRCC (**A**). GO and KEGG^32–34^ functional enrichment analysis of EYAs and the 50 neighboring genes related to the mutations of EYAs in ccRCC (**B**).

Following an analysis of genetic changes and their prognostic significance in ccRCC patients, we investigated 50 neighboring genes associated with EYAs mutations. We used the STRING database to construct an integrated network (https://string-db.org/). As showcased in Fig. 8A, the regulation of DNA metabolic process and double-strand break repair-related genes including ATM, ATR, BLM, BRCA1, BRCA2, CHEK1, CTNNB1, and DACH1 were significantly associated with EYAs mutations. With the “cluster Profiler” package in R^31^, we performed Gene Ontology (GO) and Kyoto Encyclopedia of Genes and Genomes (KEGG) pathway enrichment analyses on EYAs and their 50 most frequently altered neighboring genes, further investigating their functions (Supplementary Table 2). In Fig. 8B, BP (biological processes) like GO:0,010,212 (response to ionizing radiation), GO: 0,051,052 (regulation of DNA metabolic process), and GO: 0,006,302 (double-strand break repair) are depicted. Cellular components, including GO:0,000,781 (chromosome, telomeric region), GO: 0,098,687 (chromosomal region), and GO: 0,000,784 (nuclear chromosome, telomeric region) were significantly associated with the EYAs alterations. Furthermore, mutations of EYAs influenced molecular functions, such as GO: 0,003,684 (damaged DNA binding), GO: 0,140,097 (catalytic activity, acting on DNA), and GO: 0,003,678 (DNA helicase activity). In KEGG analysis, 3 pathways including hsa: 03,440 (Homologous recombination), hsa: 03,460 (Fanconi anemia pathway), and hsa: 04,218 (Cellular senescence) were related to the functions of EYAs mutations in ccRCC.

### Immune Infiltration Analysis of the EYAs Family in ccRCC

**Figure 9:**
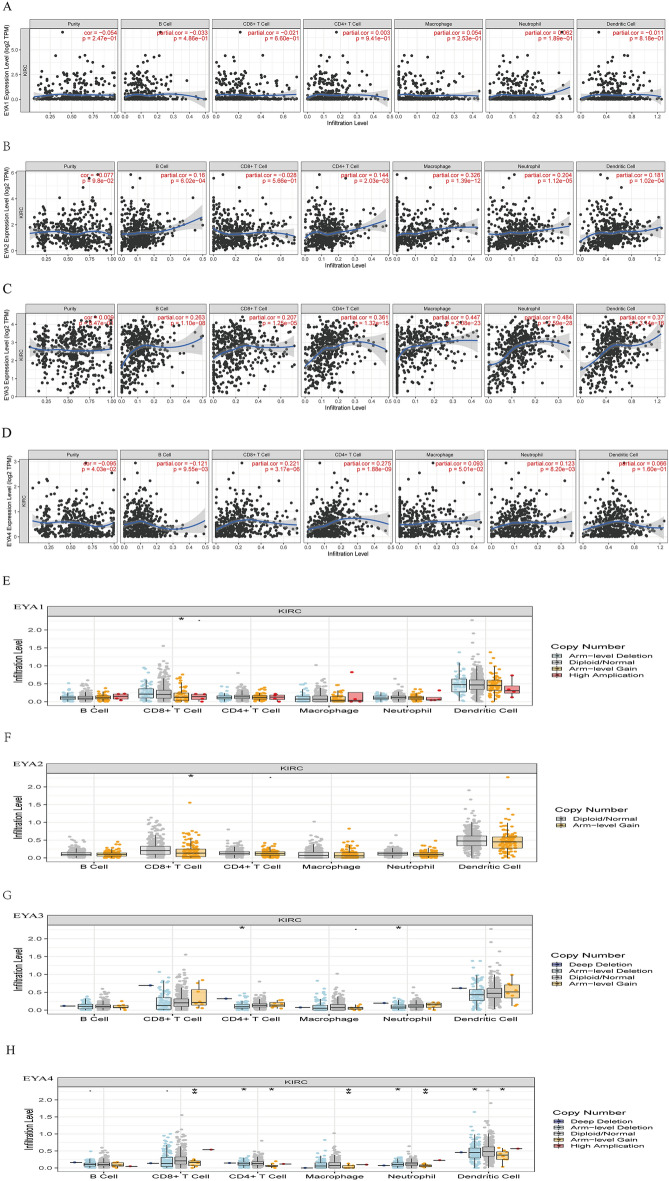
Correlation between EYAs genes and immune cell infiltration (**A**–**D**). Correlation between tumor infiltration levels in ccRCC and different somatic copy number alterations for EYAs (**E**–**H**) (TIMER). ns indicates not significant; *P* ≥ 0.05; *P < 0.05; **P < 0.01.

Using the TIMER database, correlations between immune infiltration and genes were conducted. The tumor microenvironment is comprised of a wide range of immune cells that are infiltrated around the tumor tissue^35^. As the results presented in Fig. 9A–D, there was no relationship between EYA1 and infiltration of immune cells. EYA2 showed a positive correlation with the infiltration of B cells, CD4 + T cells, macrophages, neutrophils, and dendritic cells. EYA3 showed a positive correlation with the infiltration of B cells, CD8 + T cells, CD4 + T cells, macrophages, neutrophils, and dendritic cells. EYA4 was positively correlated with the infiltration of B cells, CD8 + T cells, CD4 + T cells, and neutrophils.

Subsequently, we utilized TIMER to explore the association between different somatic copy number alterations and immune cell infiltration in ccRCC specimens. We then focused on examining the SCNA of EYAs. Our findings revealed that SCNA of EYA1/2 had a notable correlation with the levels of infiltrating CD8 + T cells, while SCNA of EYA3 was significantly linked with infiltrating levels of CD8 + T, macrophages, CD4 + T, neutrophils, and dendritic cells. Meanwhile, the SCNA of EYA2 showed a positive correlation with neutrophils and CD4 + T cells (Fig. 9E–H). Collectively, EYAs members were closely associated with the immune response in patients with ccRCC.

**Figure 10:**
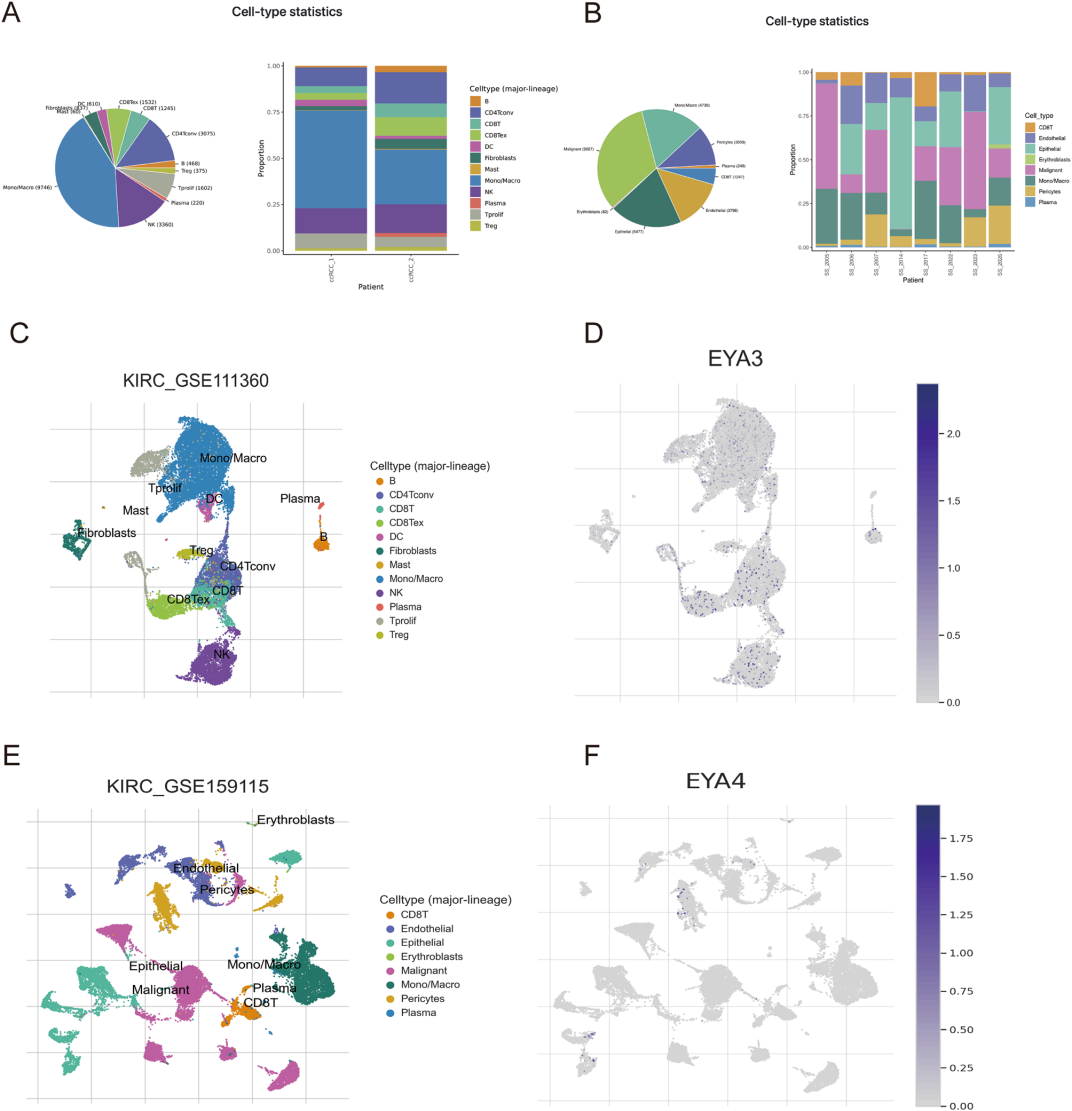
Percentage of KIRC_GSE111360 (**A**) cell numbers and KIRC_GSE159115 (**B**). Cell type distribution of KIRC_GSE111360 (**C**) and KIRC_GSE159115 (**E**). Distribution of cells expressing EYA3 (**D**) and EYA4 (**F**).

In our chosen GEO dataset (GSE111360), it is evident that the number of Mono/Macro cells was higher in both patients, followed by NK cells (Fig. 10A). Then, cells expressing the EYA3 gene were distributed in most immune cells, with a relatively high distribution in CD4 Tconv cells, CD8 T cells (Fig. 10C,D). In the GEO dataset (GSE159115), malignant cells were the most distributed, followed by epithelial cells and Mono/Macro cells (Fig. 10B). Cells expressing the EYA4 gene, although less distributed in this dataset, are more clearly distributed on CD8 T cells (Fig. 10E,F).

### Verification of the drug sensitivity of the EYAs family

**Figure 11:**
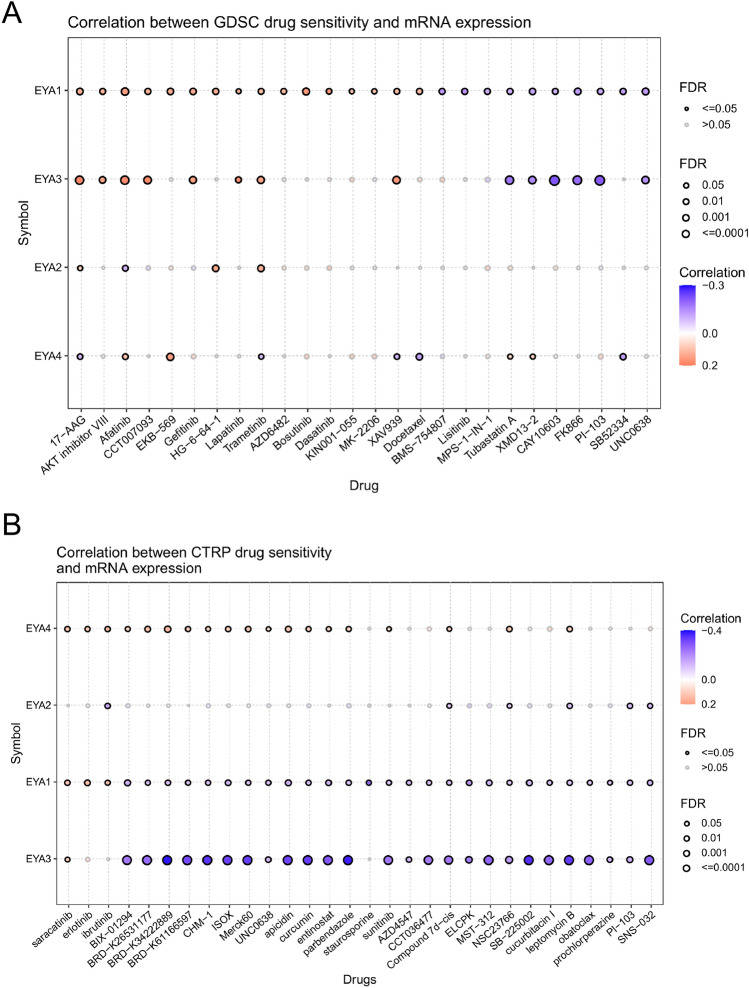
Drug sensitivity of EYA1/2/3/4 genes from GSCA. The bubble plot exhibits the correlations between gene expression and FDA-approved drugs. The positive Spearman correlation coefficients indicate that high gene expression is resistant to drugs via Genomics of Drug Sensitivity in Cancer (GDSC) and Cancer Therapeutics Response Portal (CTRP) (**A**, **B**).

Through the GSCALite platform, we found that high expression of EYA1/3 negatively correlated with some drugs and positively with some drugs, while EYA2/4 played little role in medicine resistance based on the GDSC database (Fig. 11A). The expression of EYA1/3 showed negative correlations with drugs or small molecules and the expression of EYA4 exhibited negative correlations with small molecules according to CTRP (Fig. 11B). The results showed that EYA1/3/4 expression correlated with drug resistance, thus suggesting that EYA1/3/4 could be used as biomarkers for determining drug sensitivity.

### Correlation between EYAs expression and methylation in ccRCC

In recent research, epigenetic factors, such as DNA methylation, have been demonstrated to regulate gene expression and play a key role in cancer development and progression. Our next step was to investigate the correlation between EYAs expression and methylation using the GSCALite and UALCAN databases. As presented in Supplementary Fig. 1A–D, EYA3/4 members showed remarkably decreased methylation levels in ccRCC tissues while no relationship between the expression of EYA1 and methylation was found based on the UALCAN database. As presented in Supplementary Fig. 1E, EYA1/3/4 expression, and methylation exhibited a negative correlation, while EYA2 expression and methylation showed no correlation according to the GSCALite database. Taken together, it was necessary to explore the change in the methylation status of EYAs genes in ccRCC. Further investigation into the mechanism behind EYA methylation might prove beneficial for the treatment of patients with ccRCC.

## Discussion

Due to their intricate composition, the EYA proteins significantly impact tumor progression through various mechanisms. This is attributed to their possession of two distinct phosphatase domains and a transcriptional activation domain. During early embryogenesis, the EYA proteins were first recognized as crucial co-activators of the six families of homeoprotein, which are essential for the development of various organs^36^. The EYA protein's tyrosine phosphatase activity is vital for fly eye development, while in vertebrates, EYA promotes DNA damage repair following genotoxic stress^37,38^. Studies have demonstrated that the tyrosine phosphatase activity of EYAs is linked to the regulation of various cellular phenotypes, which is closely associated with tumor progression^39^. Distinct cellular functions, biological roles, and disease states have been associated with each of these activities. There is growing evidence that dysregulation of the EYA factor is associated with many cancers, indicating that EYAs were involved not only in tumor cell and tumorigenesis proliferation but also in tumor metastasis^18,19,40,41^. There are many biomarkers for renal clear cell carcinoma, for example, Ning Yi Yap et al. showed in a CD14 immunohistochemical assay of 88 pairs of renal clear cell carcinoma and their adjacent non-cancerous kidney tissues that CD14-positive tumors and immune cells were strongly associated with cancer progression^42^. Meanwhile, CA9 also showed good prediction in mall solid renal mass (≤ 4 cm)^43^. While some members of the EYAs family have been confirmed to play an important role in ccRCC, the distinct roles of EYAs family members remain undefined. Further bioinformatics analysis of ccRCC has yet to be performed. In the present study, we examined EYAs mRNA expression, mutation, and prognostic values in ccRCC for the first time. This study aims to improve treatment design and enhance prognosis accuracy for patients bearing ccRCC.

Over-expression of EYA1 mRNA was found in ccRCC tissues and mRNA expression of EYA2/3/4 expressed low in ccRCC patients. Moreover, it was reported that the mRNA expression of EYA2 was generally reduced in colorectal cancer and higher EYA2 expression might predict a more favorable prognosis^44^. Research has shown that the upregulation of cyclin proteins and ERK signaling is promoted by the interaction between overexpressed EYA2 and Six1, which leads to increased proliferation and invasion of astrocytoma cells^45^. The above research indicates that each of the EYAs molecules has a specific role to play in different cancer types. The same EYAs molecule acts either as an oncogene or tumor-suppressive gene based on tumor heterogeneity.

The EYA1, which is an essential member of the Retinal Determination Gene Network (RDGN), has been implicated in the promotion of various cancers^46^. By activating cyclin D1, expressing EYA1 exogenously contributed to breast tumor growth and induced the properties of cancer stem cells (CSCs)^47^. Zhang et al.^16^ concluded that EYA1 could promote migration and invasion via activating FNDC3B as a cancer-promoting gene in HCC and might become a poor predictor for HCC patients. However, some scholars found that the role of EYA4 showed the opposite result compared with the role of EYA1 in HCC. Zhu et al. suggested that EYA4 suppressed HCC tumor cell growth by repressing MYCBP by dephosphorylating β‐catenin, S552, and HCC patients with high expression of EYA4 had significantly longer DFS and OS compared to HCC patients with low expression of EYA4^48^. In this study, the expression of EYA1 was upregulated in ccRCC tissues compared with normal renal tissues, while EYA4 showed the opposite expression based on our experimental results. In agreement with our analysis, co-expression between the EYA1 and EYA4 is negative in ccRCC (Fig. 7C). In addition, we found that high expression of EYA1 in ccRCC patients was significantly associated with clinicopathological parameters, including pathological stage, tumor grade, shorter OS, and PFI (progress free interval). The research above indicated that the role of EYA1/4 in ccRCC might be the same as the role of EYA1/4 in HCC, which served as a potential oncogene and a tumor suppressor, respectively. Additionally, the independent prognostic value for overall survival (OS) of ccRCC patients is attributed to the expression of EYA1.

Eyes absent homolog 2 (EYA2), a transcriptional activator that plays a critical role in organ development, has been observed to exhibit abnormal regulation in various human tumors^44,45,49^. Numerous studies have demonstrated that EYA2 is overexpressed in several cancer types, such as breast, ovarian, and lung cancer, and its upregulation is linked to a poorer prognosis^18,19,50^, while the expression of EYA2 in colorectal cancer was generally reduced, and the expression of EYA2 indicated a better prognosis^44^. Lung cancer cells have a hypomethylated EYA2 gene, resulting in overexpression of EYA2^44^. In our study, EYA2 showed a remarkably high methylation level in ccRCC tissues, while the EYA2 mRNA expression was low in ccRCC tissues and was not associated with the prognostic stage or any of the tumor grades. The reason for this phenomenon might be that the mRNA level of EYA2 might be regulated by miRNAs^49,51^. Aberrant methylation in the promoter region contributes to the downregulation of genes in tumors. According to Vincent et al., EYA2 was silenced in pancreatic cancer cell lines due to the methylation of its promoter^52^, which was consistent with the promoter methylation of EYA3/4 in ccRCC. Although some scholars found EYA2 was found to be hypermethylated in breast cancer tissues compared with adjacent normal tissues^53^, EYA2 promoted breast cancer progression in some studies^54,55^, suggesting that methylation of EYA2 might not be the primary cause of breast cancer progression to a great extent.

As one of the EYA family of proteins, currently, there are few research reports on the EYA3 gene in cancer. Some studies showed that the EYA3 gene was frequently deleted in certain pancreatic ductal adenocarcinomas (PDAC)^56^. In the study, we confirmed that the expression of EYA3 was higher in normal tissues than in kidney cancer tissues. We found that higher mRNA expression of EYA3 was significantly associated with longer OS and PFI of ccRCC, suggesting that EYA3 could function as a tumor suppressor. Furthermore, multivariate Cox regression analyses found low EYA3 expression to be an independent prognostic factor in ccRCC. Our study found that EYA3 was positively correlated with the infiltration of immune cells in ccRCC, suggesting the participation of EYA3 in the regulation of cancer immunity. The significant role of immune cells in regulating tumor growth has been well established, with infiltrating immune cells surrounding tumors gaining recognition as critical regulators^57,58^. It had been reported that EYA3 enhanced breast tumor growth via regulating cytotoxic T cells and was associated with increased numbers of infiltrated CD8 + T cells^59^. Together with other findings discussed above, collectively, these findings point to EYA3 being a promising prognostic and therapeutic target for patients bearing ccRCC.

The EYA4 gene was first identified by Borsani et al. in 1999 and has been reported to be dysregulated in many types of human cancer^60^. Hypermethylation and reduced expression of EYA4 have been observed in both major subtypes of non-small cell lung cancer and even in the initial stages of the disease^61^. The expression of EYA4 in PDAC tissues is significantly reduced, and PDAC patients with downregulated expression of EYA4 have a shorter OS time^62^. In addition, the EYA4 gene has also been identified as a promising tumor suppressor gene for colorectal cancer since it controls DKK1 upregulation and blocks the Wnt signaling pathway^63^. Consistent with these findings, this study showed that hypermethylation and low expression of EYA4 have been detected in ccRCC, and a significant association was found between higher EYA4 mRNA expression levels and longer OS and PFI of ccRCC. As the tumor grade increased, the mRNA expression level of EYA3/4 decreased. We observed that a positive correlation was found between EYA4 expression and immune cell infiltration, whereas a negative correlation was found between EYA4 expression and small molecule levels. The results of the study affirmed that EYA4 might serve as a crucial therapeutic target and prognostic biomarker for ccRCC. The findings also suggested that EYA4 functioned as a tumor suppressor.

Our study comprehensively investigated the expression and prognostic significance of EYA1/2/3/4 genes in ccRCC. Moreover, we conducted experiments to confirm the expression of EYAs in ccRCC tissues. The study provides a detailed understanding of EYAs as potential biomarkers and targets for the treatment of renal cancer. Despite our study's contribution to demonstrating the prognostic value of mRNA expression of EYA1/3/4 in ccRCC, it has certain limitations. Firstly, further investigation with larger sample sizes is necessary to confirm and explore the potential clinical utility of EYAs members as prognostic factors for ccRCC. Additionally, a more in-depth analysis of EYAs is required to validate our findings. An additional limitation of our study is the lack of exploration into the potential mechanisms underlying the distinct roles of EYAs in ccRCC. Further studies were warranted to investigate the underlying molecular mechanisms in ccRCC.

## Conclusions

Our study focused on investigating the expression of EYAs and their clinical significance in ccRCC. The results showed that overexpression of EYA1 could contribute to ccRCC development, while low expression of EYA3/4 might have a tumor-suppressive effect. Thus, EYA1/3/4 proteins could serve as potential targets for kidney cancer therapy and prognostic markers for improving patient survival and accuracy.

## Supplementary Information

Supplementary Information.

## Data Availability

The datasets used and/or analysed during the current study available from the corresponding author on reasonable request.
