# Supplementary material for: The value of EYA1/3/4 in clear cell renal cell carcinoma: a study from multiple databases
Source: Sci Rep. 2023 May 8;13:7442. doi: 10.1038/s41598-023-34324-3 (PMC10167363; doi:10.1038/s41598-023-34324-3)

**
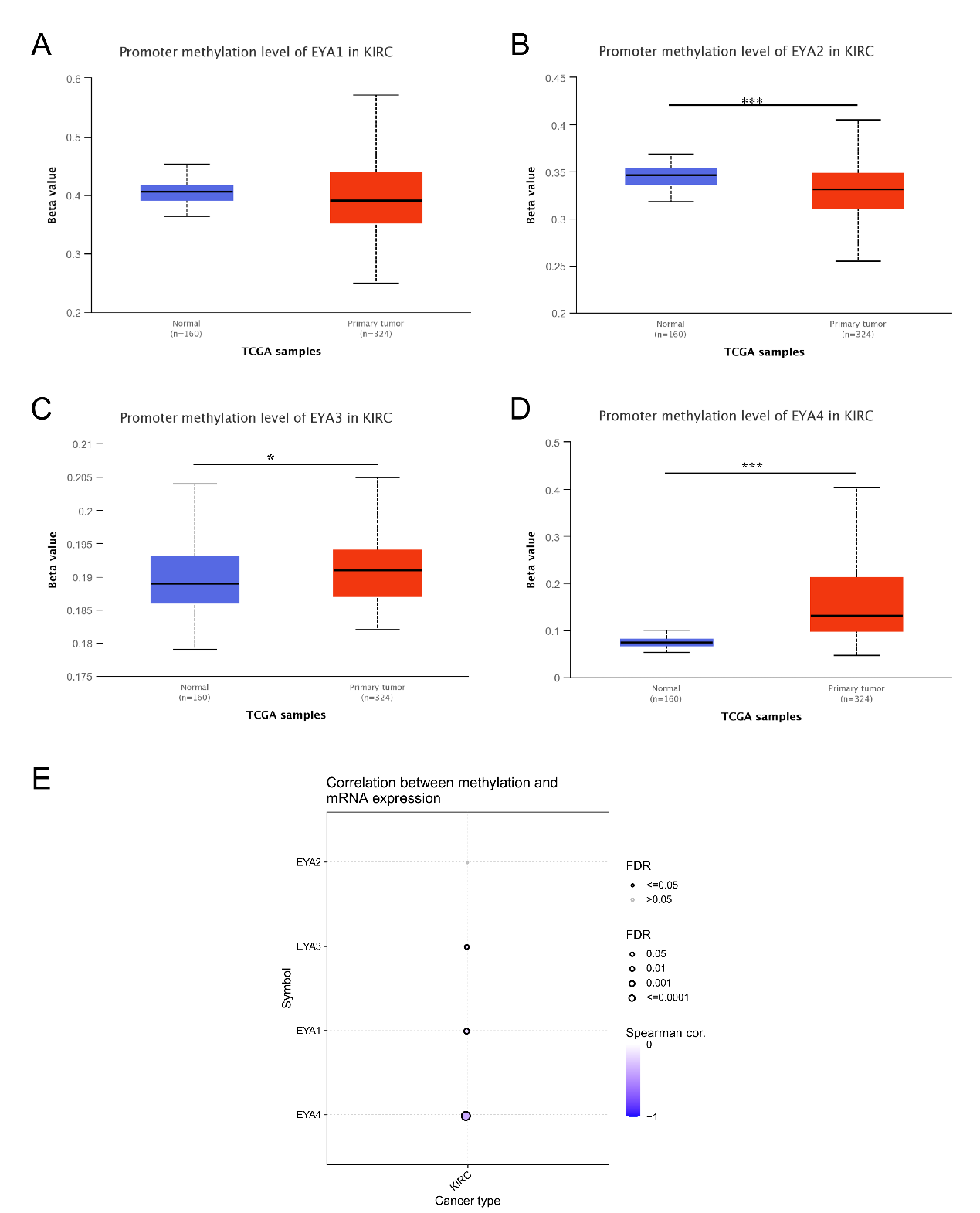
**

**Supplementary Fig 1**. Correlation between EYAs expression and methylation levels based on UALCAN (A-D). Spearman correlation between methylation and expression of EYAs based on GSCALite database (E). ns indicates not significant; * *p* < 0.05; *** *p* < 0.001.

**Supplementary Table 1**. univariate and multivariate analyses of factors contributing to the overall survival of ccRCC patients.


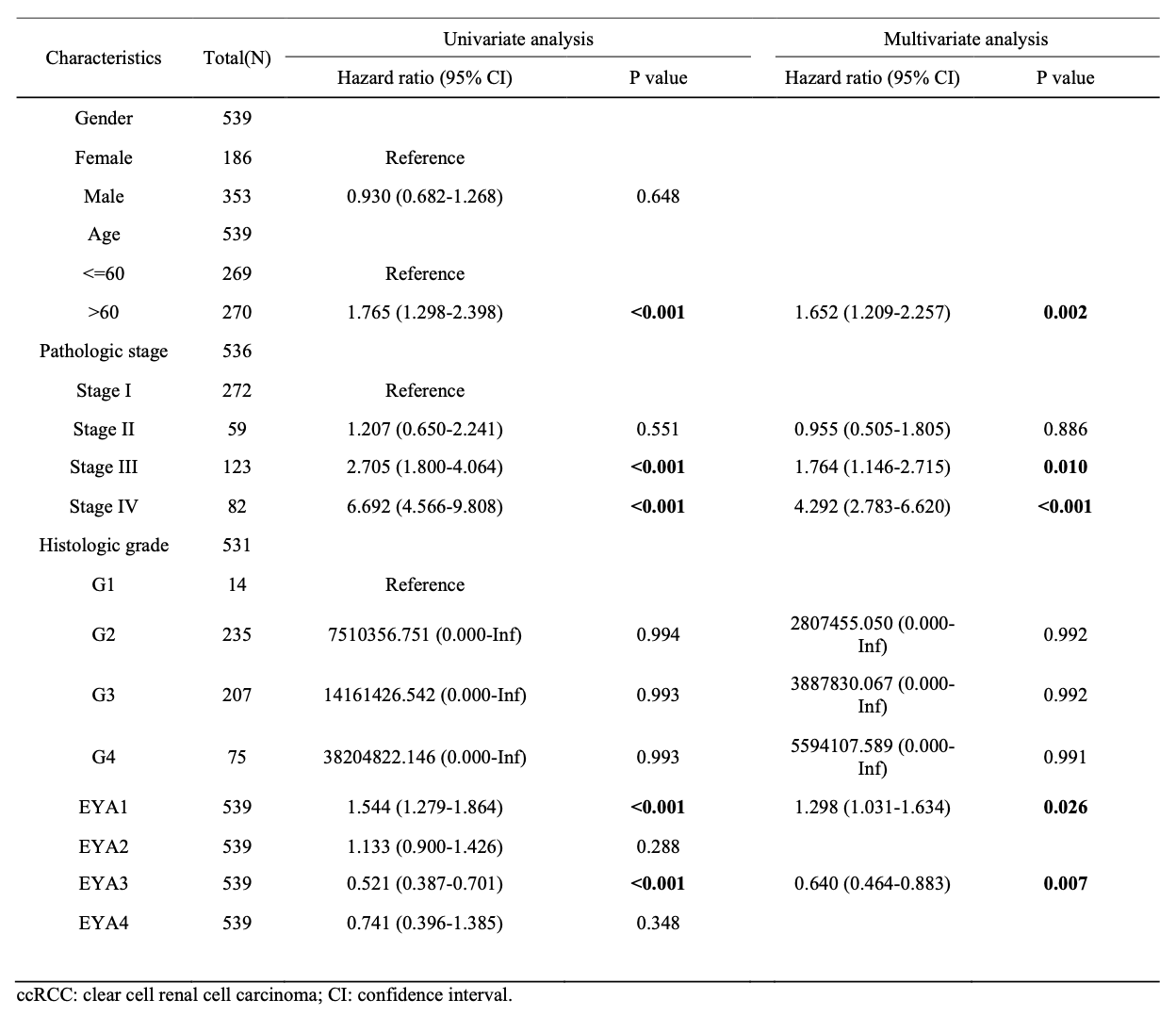


**Supplementary Table 2**. GO and KEGG functional enrichment analyses of EYAs and their 50 associated neighbor genes.


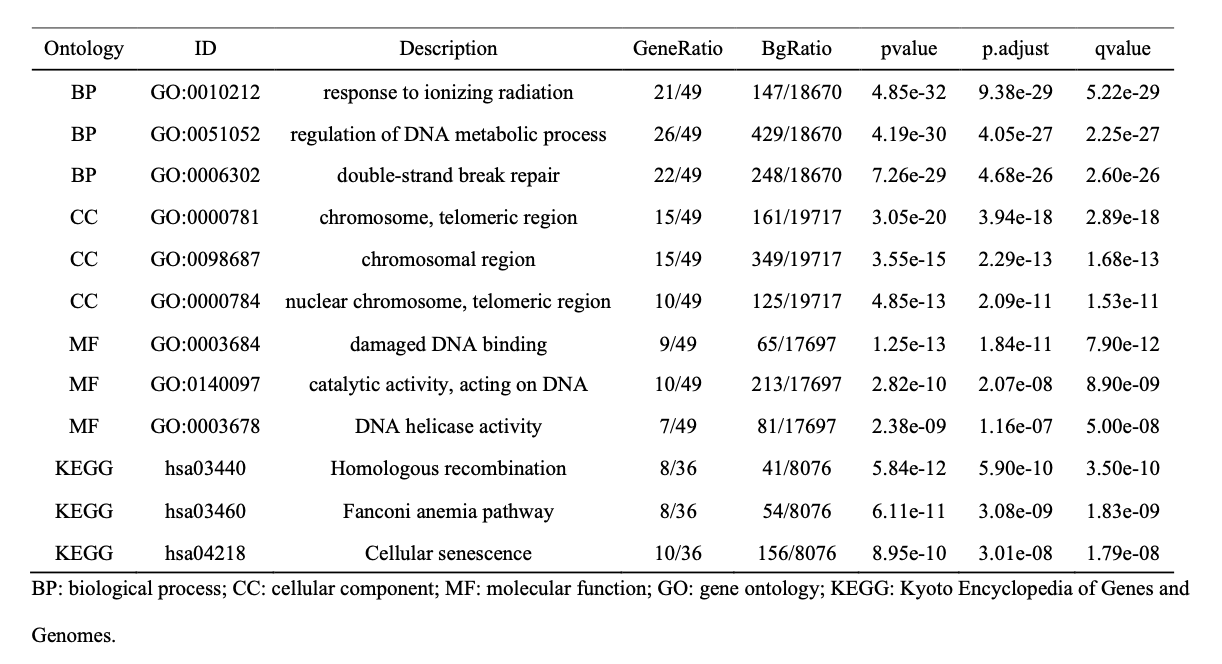

Supplement: Supplementary file 1 — Supplementary Information. [file 41598_2023_34324_MOESM1_ESM.docx]
